# Accelerometer-Measured Diurnal Patterns of Sedentary Behavior among Japanese Workers: A Descriptive Epidemiological Study

**DOI:** 10.3390/ijerph17113814

**Published:** 2020-05-27

**Authors:** Sayaka Kurosawa, Ai Shibata, Kaori Ishii, Mohammad Javad Koohsari, Koichiro Oka

**Affiliations:** 1 Graduate School of Sport Sciences, Waseda University, Saitama 359-1192, Japan; 2 Faculty of Health and Sport Sciences, University of Tsukuba, Ibaraki 305-8574, Japan; shibata.ai.ga@u.tsukuba.ac.jp; 3 Faculty of Sport Sciences, Waseda University, Saitama 359-1192, Japan; ishiikaori@waseda.jp (K.I.); javadkoohsari@aoni.waseda.jp (M.J.K.); koka@waseda.jp (K.O.); 4 Behavioural Epidemiology Laboratory, Baker Heart and Diabetes Institute, Melbourne, VIC 3004, Australia; 5 Melbourne School of Population and Global Health, The University of Melbourne, Melbourne, VIC 3010, Australia

**Keywords:** accelerometer, sitting time, sociodemographic correlates, daily patterns

## Abstract

Increased sedentary behavior (SB) can adversely affect health. Understanding time-dependent patterns of SB and its correlates can inform targeted approaches for prevention. This study examined diurnal patterns of SB and its sociodemographic associations among Japanese workers. The proportion of sedentary time (% of wear time) and the number of breaks in SB (times/sedentary hour) of 405 workers (aged 40–64 years) were assessed using an accelerometer. SB patterns and sociodemographic associations between each time period (morning, afternoon, evening) on workdays and nonworkdays were examined in a series of multivariate regression analyses, adjusting for other sociodemographic associations. On both workdays and nonworkdays, the proportion of sedentary time was lowest in the morning and increased towards evening (*b* = 12.95, 95% CI: 11.28 to 14.62; *b* = 14.31, 95% CI: 12.73 to 15.88), with opposite trend for breaks. Being male was consistently correlated with SB. Other sociodemographic correlates differed depending on time-of-day and day-of-the-week. For instance, desk-based workstyles and urban residential area were associated with SB during workday mornings and afternoons, being single was related to mornings and evenings, workdays and nonworkdays. Initiatives to address SB should focus not only on work-related but time-of-day contexts, especially for at-risk subgroups during each period.

## 1. Introduction

Longer time spent in sedentary behavior (SB), which is distinct from lack of physical activity, is associated with poor health outcomes, such as the higher risk of all-cause mortality, type 2 diabetes mellitus, some types of cancer, and cardiovascular diseases [1]. The recent technological revolution has promoted automatization and computerization of work, resulting in a reduction of physical demand and increase in sedentary time at work [2]. For example, the average working time among Japanese workers was approximately eight hours per day in 2017 [3]. Thus, workers are one of the at-risk populations for longer time spent in SB, and it is necessary to focus on reducing worker’s SB as a public health strategy.

Characterizing SB patterns and their sociodemographic associations may play a key role in planning effective intervention strategies such as targeting particular SB patterns and subgroups that require intervention [4]. Among workers, most of the previous studies focused on the total amount of daily SB and work-related timeframes (e.g., work/nonwork days or times) [5,6,7,8,9]. While the work-related timeframe is essential to understand workers’ SB, examining diurnal patterns (e.g., morning, afternoon, evening) may also provide useful information on time-dependent SB patterns. Only a small number of studies have graphically illustrated the diurnal pattern of desk-based SB through the course of the day for weekdays and weekends [10,11,12,13]. For example, Smith et al. [12] graphically demonstrated that the greatest amount of time desk-based workers’ spend sitting occurred between 09:00–12:00, 14:00–17:00, and 20:00–23:00 on the weekdays, and between 18:00–23:00 on the weekend.

Nevertheless, to our knowledge, there is no previous research comparing the quantitative differences in the diurnal pattern of sedentary time across different subgroups of workers. Understanding the diurnal patterns of SB and their sociodemographic associations provide useful information to identify the potential or suitable timing for reducing SB throughout the day among workers. Therefore, this study examined diurnal patterns and their sociodemographic correlates in objectively measured SB among Japanese workers.

## 2. Materials and Methods

### 2.1. Participants

**Figure 1:**
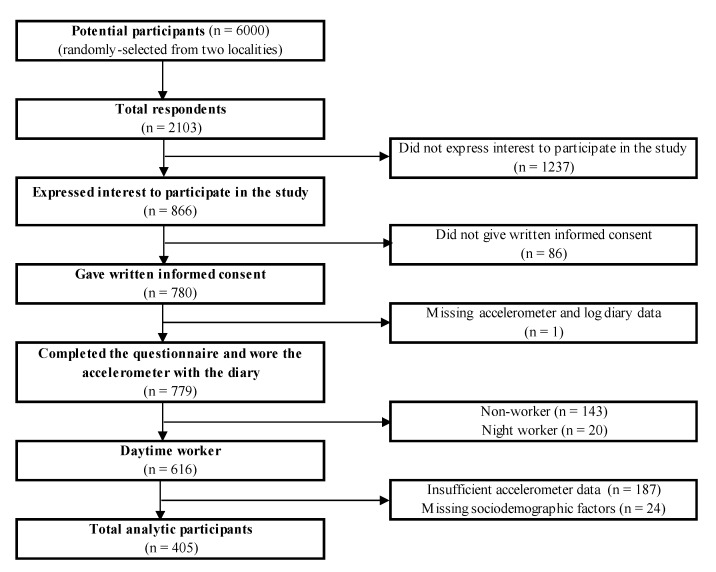
The flowchart of participant recruitment.

This study used a cross-sectional survey distributed between July and December 2013 and from April 2014 to February 2015, as a part of a project aimed at examining the associations between social and urban design attributes and SB among middle-aged Japanese adults. A total of 6000 residents, aged 40–64 years living in two Japanese cities (Koto Ward: Tokyo metropolitan area, a higher-dense area with a population of 480,271 in January 2013; Matsuyama City: a local city of Shikoku region, a lower-dense area with a population of 517,838 in June 2014), were randomly selected from the residential registries of each area, stratified by sex and age group. For the selection of study sites, two cities with similar population size but typically different population density (approximately ten times difference) were chosen from popular cities in Japan. An invitation letter was sent to all potential participants. About two weeks later, a reminder letter was sent to the nonrespondents. A questionnaire and an accelerometer (with a log diary) were sent to those who agreed to participate in this study. Participants were asked to complete the questionnaire and the diary and return them within two weeks. A reminder letter was sent to nonresponders up to three times, and a 1000-yen book voucher was offered to those who completed the questionnaire and wore the accelerometer with the diary. A total of 779 participants completed the survey (90.0% of the initially approached sample). Of these, day-time workers were included for this study (n = 616), because the majority of workers in Japan work during day-time and have different work and sleep patterns from night workers. Those who had insufficient accelerometer data (n = 187) or missing or invalid data for sociodemographic factors (n = 24) were excluded. The final sample size was 405. The flowchart of participant recruitment is shown in Figure 1. All participants gave written informed consent. This study was approved by the Ethics Committee of Waseda University (# 2012-269), Japan.

### 2.2. Measures

#### 2.2.1. Sedentary Behavior and Physical Activity

SB and physical activity were measured by a triaxial accelerometer (Active style Pro HJA-350IT; Omron Healthcare Co. Ltd., Kyoto, Japan). This device was reported to have high validity [14] and reliability [15]. The participants were asked to wear the accelerometers during waking hours for seven consecutive days and to remove them during any water-based activities or contact sports. Nonwear time was defined as intervals of at least 60 consecutive minutes of no activity (0.9 or less Metabolic Equivalents (METs)) [14], with allowance for up to two minutes of observations of some limited movement (≤ 1.0 METs) within these periods [16]. Due to sparse data during the early morning and late evening, time spent in SB and engaged in physical activity were calculated between 06:00–23:59 and each period of the day (morning (06:00–11:59), afternoon (12:00–17:59), evening (18:00–23:59)) on workdays and nonworkdays. Days containing at least 10 h of wear time were regarded as valid [16]. Using the log diary, participants were asked to report the time when they put on and took off the accelerometer. The log diaries were checked along with accelerometer data to verify the time periods during which participants wore the accelerometer and days of the week. Those participants who completed at least four valid days, including at least one nonworkday, were included. Finally, participants with a minimum of 25% valid hours of wearing time in each of the three time periods [17] of at least three workdays and one nonworkday were included [7].

Three measures of SB and physical activity were designated for the total and each time period—sedentary, light-intensity physical activity (LPA), and moderate-to-vigorous physical activity (MVPA) time (minutes/period), the proportion of sedentary, LPA and MVPA time (% of wear time), and the number of breaks in SB (times/sedentary hour). SB, LPA, and MVPA were defined as wear time for any activity with an accelerometer-estimated intensity of ≤ 1.5, 1.5 and < 3.0, and 3.0 or more METs, respectively [18]. A break was defined as a period of nonsedentary minutes in between two sedentary bouts [19].

#### 2.2.2. Sociodemographic Factors

Sex and age were obtained from residential registries. Height, weight, marital status (single, married), household income (< 5 million yen, ≥ 5 million yen), educational attainment (high school or less, two years of college or higher education), employment status (full-time, part-time), workstyle (desk-based, non-desk-based), smoking status (smoker, nonsmoker), and alcohol consumption (≤ 1–3 times/month, ≥ 1 time/week) were reported by participants. Body mass index (BMI; kg/m^2^) was calculated from self-reported height and weight. The residential area was classified into urban (Koto) and suburban (Matsuyama).

### 2.3. Statistical Analyses

All analyses were separately conducted for workdays and nonworkdays. Overall average values of SB, LPA, and MVPA during 6:00–23:59 (whole day) and each period (morning, afternoon, and evening) were reported. The diurnal differences in the proportion of sedentary time (% of wear time) and the number of breaks (times/sedentary hour) were examined using the multilevel mixed-effects linear regression models (time periods as level 1 and individuals as level 2) with random intercepts, adjusting for sociodemographic variables. Differences in the proportion of sedentary time (% of wear time) and the number of breaks (times/sedentary hour) between subgroups in a whole day or each time period were examined using multiple regression analysis, adjusting for other sociodemographic variables. Interaction effects between sociodemographic variables and time period were examined using multilevel mixed-effects linear regression models with random intercepts. Stratified analysis was conducted for variables which demonstrated significant interactions. Several graphs were also created to facilitate the interpretation of the interaction effects. Statistical analyses were performed using STATA 15.0 (Stata Corp LLC, TX USA), and the level of significance was set at *p* < 0.05 for differences and *p* < 0.1 for interactions.

## 3. Results

### 3.1. Characteristics of Participants

Table 1 presents the characteristics of the final study sample of 405 participants. Mean age was 51.5 (standard deviation (SD), 7.0) years. Approximately half of the participants were women. Most participants were married; more than half had greater than or equal to 5 million yen of income, and two-thirds had graduated with two years of college or higher education. The participants were mainly full-time workers with a desk-based workstyle. A small number of participants were overweight (BMI ≥ 25 kg/m^2^), smokers and half reported alcohol consumption one or more times per week. On workdays, the average length of time wearing the accelerometer was 937.0 (69.4) minutes overall, of which 352.4 (126.7) minutes were LPA and 77.4 (47.7) minutes were MVPA. On nonworkdays, mean time wearing the accelerometer was 880.6 (90.2) minutes throughout the day, of which 313.2 (109.5) and 56.7 (36.0) minutes were LPA and MVPA, respectively. Averaged minutes of accelerometer wearing time, LPA, and MVPA on three time periods of work and nonworkdays are presented in the Supplementary Table.

### 3.2. Prevalence of Sedentary Behavior for the Whole Day and Three Time Periods

The mean (SD) sedentary time during the whole day (06:00–23:59) was 507.3 (140.2) minutes on workdays and 510.7 (127.5) minutes on nonworkdays, accounting for 54.2% (14.9%) and 58.0% (13.5%) of wearing time, respectively (Table 2). Both on workdays and nonworkdays, the average proportion of sedentary time was the lowest in the morning and highest in the evening. In the morning, the average proportion of sedentary time was 48.3% and 52.6%, respectively. These proportion were significantly higher in the afternoon (54.1%, *b =* 5.86, 95% CI: 4.19 to 7.53, *p* < 0.001; 55.6%, *b =* 2.97, 95% CI: 1.39 to 4.54, *p* < 0.001) and evening (61.2%, *b =* 12.95, 95% CI: 11.28 to 14.62, *p* < 0.001; 66.9%, *b =* 14.31, 95% CI: 12.73 to 15.88, *p* < 0.001). The difference between afternoon and evening was also significant (*b =* 7.09, 95% CI: 5.42 to 8.76, *p* < 0.001; *b =* 11.34, 95% CI: 9.76 to 12.91, *p* < 0.001). In contrast, the number of breaks on workdays and nonworkdays were the highest in the morning and lowest in the evening. The number of breaks in the morning was 12.8 times/sedentary hour and 10.4 times/sedentary hour, respectively. This number was significantly lower in the afternoon (11.0 times/sedentary hour, *b =* −1.80, 95% CI: −2.36 to −1.24, *p* < 0.001; 9.6 times/sedentary hour, *b =* −0.82, 95% CI: −1.33 to −0.31, *p* = 0.002) and evening (8.1 times/sedentary hour, *b =* −4.72, 95% CI: −5.28 to −4.16, *p* < 0.001; 7.3 times/sedentary hour, *b =* −3.16, 95% CI: −3.67 to −2.65, *p* < 0.001). Significant differences between the afternoon and evening were also observed (*b =* −2.92, 95% CI: −3.48 to −2.36, *p* < 0.001; *b =* −2.34, 95% CI: −2.85 to −1.83, *p* < 0.001).

### 3.3. Relationships Between Sociodemographic Factors and Sedentary Behavior during Whole Day and the Three Time Periods

#### 3.3.1. Workdays by Whole Day

For the whole day, the proportion of sedentary time was significantly higher in men, singles, those with higher educational attainment, desk-based workstyles, and those living in the urban area. On the other hand, women, participants who are married, those with non-desk-based workstyles, participants whose weight was in the normal range, and those participants who live in the suburban area reported higher numbers of breaks.

#### 3.3.2. Workdays by Time Period

In analyses stratified by the time periods, the proportion of sedentary time was significantly higher in men and those with desk-based workstyles, during all three time periods. Additionally, being single and living in an urban area were significantly associated with a higher proportion of sedentary time during the morning period. Those participants with higher educational attainment, individuals with a full-time job, and those living in the urban area were more likely to spend time in SB during the afternoon period. There were no additional sociodemographic attributes significantly associated with the proportion of sedentary time during the evening period. Regarding the number of breaks, woman, non-desk-based workstyles, and the suburban residential area were significantly associated with taking more breaks during both the morning and the afternoon periods. Married participants and those with normal weight status (BMI < 25 kg/m^2^) were also significantly associated with higher numbers of breaks during morning and afternoon periods, respectively. During the evening period, smokers were the only sociodemographic group that was significantly associated with more breaks.

#### 3.3.3. Nonworkdays by Whole Day

For the whole day, men, being single, and those who consumed alcohol less frequently had a significantly higher proportion of sedentary time than participants in other sociodemographic groups. The number of breaks was significantly higher in women and married individuals.

#### 3.3.4. Nonworkdays by Time Period

Analyses of nonworkdays stratified by time period showed a significantly higher proportion of sedentary time observed for men during all three time periods. Additionally, participants who were single, those with desk-based workstyles, and those who had a lower frequency of alcohol consumption had a significantly greater proportion of sedentary time during the morning period. On the other hand, there were no additional sociodemographic attributes significantly related to the proportion of sedentary time during the afternoon period. During the evening period, those whose age was ≥ 50 years and those who were single showed significantly higher proportions of the sedentary time. In terms of breaks, women and married participants had significantly more breaks during the morning period. Women, during the afternoon period, and married participants and those aged < 50 years during the evening period also had significantly more breaks, respectively.

### 3.4. Interaction Effects

**Figure 2:**
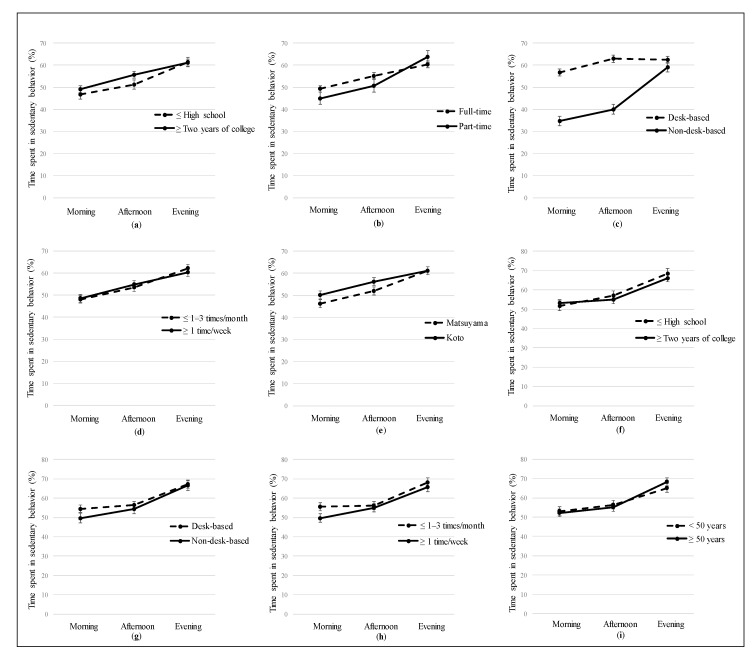
Diurnal patterns of sedentary behavior by sociodemographic group. SB—sedentary behavior. The interaction effects of the proportion of sedentary time between subgroups of each sociodemographic variable were examined using multilevel mixed-effects linear regression, and stratified analysis was conducted for variables that showed significant interactions: (**a**) Workday SB by education attainment; (**b**) Workday SB by employment status; (**c**) Workday SB by workstyle; (**d**) Workday SB by alcohol consumption; (**e**) Workday SB by residential area; (**f**) Nonworkday SB by educational attainment; (**g**) Nonworkday SB by workstyle; (**h**) Nonworkday SB by alcohol consumption; (**i**) Nonworkday SB by age. The values were expressed as means and 95% confidence intervals.

Interaction effects with diurnal patterns, on workdays, were significant for educational attainment (Figure 2a), employment status (Figure 2b), workstyles (Figure 2c), alcohol consumption (Figure 2d), and residential area (Figure 2e). The increase in the proportion of sedentary time from afternoon to evening was significantly greater in participants with lower educational attainment (*b =* 4.87; 95% CI, 1.92 to 7.82; *p* = 0.001), part-time job (*b =* 8.02; 95% CI, 4.64 to 11.40; *p* < 0.001), non-desk-based workstyles (*b* = 19.45; 95% CI, 16.38 to 22.53; *p* < 0.001), low frequency of alcohol consumption (*b* = 3.48; 95% CI, 0.72 to 6.25; *p* = 0.014), and living in a suburban area (*b* = 4.15; 95% CI, 1.28 to 7.01; *p* = 0.005).

On nonworkdays, there were significant interactions with the diurnal pattern for educational attainment (Figure 2f), workstyles (Figure 2g), alcohol consumption (Figure 2h), and age (Figure 2i). Significantly greater SB increases from morning to afternoon were observed for participants with lower educational attainment (*b* = 3.35; 95% CI, 0.02 to 6.69; *p* = 0.049), non-desk-based workstyles (*b* = 2.96; 95% CI, −0.31 to 6.22; *p* = 0.076), and high frequency of alcohol consumption (*b* = 4.66; 95% CI, 1.55 to 7.77; *p* = 0.003). A significant increment in the proportion of sedentary time was observed from afternoon to evening for participants who were aged ≥ 50 years (*b* = 4.67; 95% CI, 1.54 to 7.80; *p* = 0.003).

## 4. Discussion

The present study examined the diurnal patterns of SB and its associations with sociodemographic attributes among Japanese workers. This study found that the proportion of sedentary time among day-time workers was lowest in the morning, increased over the time periods, and was the highest in the evening on both workdays and nonworkdays. This trend was less clear on nonworkdays due to the higher proportion of SB during the morning period. This is similar to some previous studies which have shown such a trend by the graphical illustrations of diurnal SB patterns among workers [10,11,12,13]. There were no previous studies identifying diurnal SB patterns in worker populations with quantitative analyses. Only a limited number of studies exist examining the differences in the work context, and they have found that SB during work hours was higher than those during nonwork hours [5,10,13]. The current findings imply that SB could be influenced by not only the work context but also the time period of the day in the worker population.

Our findings showed that even during nonworking (leisure) time, the patterns of SB were different—the least SB was observed in the morning, whereas most were in the evening. These patterns may be partly due to the interaction of physical and psychological conditions (less fatigued and more energetic in the morning after sleep), basic lifestyle (engaging in a household, social, and working tasks by daylight), and social structure (scheduled working, business, or store hours), which could be affected or set by general human circadian rhythms [20,21]. This interpretation may be confirmed by interaction effects found in this study—even though the patterns of SB during the morning and afternoon periods were different between subgroups in some of the sociodemographic associations, they reached approximately the same levels by the evening. On nonworkdays, this timing seems to be even earlier, in the afternoon. Considering such daily patterns in behavior and physical and psychological functions generally regulated by the circadian system, an effort to sleep earlier (to replace SB with equal amounts of sleep) might be a more feasible and beneficial approach to reduce SB rather than breaking up SB at night among the worker population, in addition to workplace interventions (e.g., sit-stand workstations for desk-based workers) conducted in previous studies [4,22,23,24,25]. Two previous studies found that sleeping less (more awake time) was associated with increased screen time (e.g., TV viewing and computer use), which is one of the sedentary domains [26,27]. Avoiding or decreasing screen time at night may have double health benefits from reducing SB and improving sleep quality as well as alertness while awake [28], especially for at-risk subgroups in the morning.

This study also demonstrated different associations in different sociodemographic attributes for each time period of SB. Regardless of the time of day and days of the week, men spent more time in SB and took fewer breaks than women throughout the day. This may reflect the difference in social and domestic roles by sex in Japan. The social situation for Japanese women, even if they are working, is characterized by a higher engagement rate for household tasks and childcare [29], which may lead them to break up SB and to be less sedentary. Additionally, it has been reported that Japanese men have long commuting and working hours [29], which may limit their opportunities for being physically active.

Regarding mornings and evenings, which are mostly categorized as leisure time, marital status was consistently associated with total sedentary time and breaks (except during the evening on workdays). These results may reflect the social roles as a parent and partner. Single adults have higher levels of sedentary leisure activities [30,31], which could be due to fewer domestic responsibilities and greater free time. Also, workers aged 50 years or older had a longer total sedentary time and fewer breaks on nonworkday evenings, which may be partly due to decreased social and parenting activities [32,33], or increased fatigue [34]. These results may indicate that sociodemographic correlates of SB are largely related to social and domestic roles during the morning and evening, in addition to work-related attributes of longer SB such as workstyles [10].

During the workday afternoon, persons with desk-based workstyles, those with higher educational attainment, those in full-time employment, and those living in a urban residential area were related to longer time spent in SB and/or fewer breaks. These results indicate that the SB on the workday afternoon (most of which is working time) could be more influenced by work-related sociodemographic attributes. Furthermore, there are previous studies that reveal positive associations of occupational sedentary time with office workers [10] and full-time employment [35]. White-collar workers [9,35] and those having a private office [36], also found in the previous studies as correlates of higher occupational SB, are partly but closely related to educational attainment. Thus, it may be necessary to target full-time and desk-based workers in Japan, similar to many previous SB intervention studies conducted in Australia and Western countries [4]. Also, the differences in SB and breaks between two residential areas may be related to the work-related sociodemographic attributes such as the kind of occupation. The Matsuyama city had a higher proportion of those who are engaged in “agriculture and forestry” or “medical, healthcare and welfare” than the Koto ward [37]. This study, however, did not directly examine occupation. Further study would need to consider more specific work-related attributes such as occupation and job position. Using sociodemographic factors that were not work-related, this study found that higher BMI was associated with fewer breaks, consistent with previous studies [35]. Thus, approaches aiming to break up prolonged sedentary time may be beneficial in overweight and obese workers.

This study had several limitations. First, having a cross-sectional design, this study is unable to determine causal relationships between variables. Second, the accelerometer devices used in this study were unable to measure water-based activity or activity that involves the movements of legs and arms and to distinguish sitting or static standing postures accurately. Finally, since there were variations in participants’ accelerometer wearing time (due to different times of waking up or going to sleep), this study cannot describe the diurnal variation of absolute time spent in SB. Future studies can also conduct cluster analysis to identify distinct subgroups of individuals with similar characteristics of diurnal sedentary patterns.

This was the first study, to our knowledge, to explore the diurnal patterns of objectively measured SB among workers and their sociodemographic associations. Another strength was the use of objectively measured SB that enabled us to investigate the accurate proportion of sedentary time and breaks during each time period.

## 5. Conclusions

In summary, the proportion of sedentary time was lowest in the morning (48.3%; 52.6%) and highest in the evening (61.2%; 66.9%) both on workdays and nonworkdays, with an inverse trend for breaks. Although being male was a consistent factor related to SB and breaks throughout the day and week, associations between other sociodemographic attributes and these SB measures differed depending on time periods of the day and the days of the week. SB and/or breaks seem to be mainly associated with work-related attributes such as workstyle, educational attainment, and employment status on workday afternoons, whereas social and domestic-related attributes such as marital status are related to mornings and evenings of workdays and nonworkdays.

## Figures and Tables

**Table 1:** Basic characteristics of study participants (n = 405).

	n	%
Age, years (mean, SD)	51.5	7.0
< 50 years	171	42.2
≥ 50 years	234	57.8
Sex: women	229	56.5
Marital status: married	321	79.3
Household income		
< 5 million yen	166	41.0
≥ 5 million yen	239	59.0
Educational attainment		
≤ High school	143	35.3
≥ Two years of college	262	64.7
Employment status		
Full-time	307	75.8
Part-time	98	24.2
Workstyle		
Desk-based	249	61.5
Non-desk-based	156	38.5
Body Mass Index, kg/m^2^ (mean, SD)	22.4	3.1
< 25	399	83.7
≥ 25	66	16.3
Smoking status: smokers	56	13.8
Alcohol consumption		
≤ 1–3 times/month	203	50.1
≥ 1 time/week	202	49.9
Residential area		
Matsuyama	199	49.1
Koto	206	50.9
Accelerometer data (mean, SD)		
Wear time (minutes)		
Workdays	937.0	69.4
Nonworkdays	880.6	90.2
SB (minutes)		
Workdays	507.3	140.2
Nonworkdays	510.7	127.5
LPA (minutes)		
Workdays	352.4	126.7
Nonworkdays	313.2	109.5
MVPA (minutes)		
Workdays	77.4	47.7
Nonworkdays	56.7	36.0

LPA, light-intensity physical activity; MVPA, moderate-to-vigorous intensity physical activity; SB, sedentary behavior; SD, standard deviation.

**Table 2:** Patterns of objectively measured sedentary behavior.

	Workdays												Nonworkdays											
	All			Morning			Afternoon			Evening			All			Morning			Afternoon			Evening		
	Mean	SD		Mean	SD		Mean	SD		Mean	SD		Mean	SD		Mean	SD		Mean	SD		Mean	SD	
***SB (%)***																								
**All participants**	54.2	14.9		48.3	18.7		54.1	19.7		61.2	12.7		58.0	13.5		52.6	17.6		55.6	16.8		66.9	14.1	
**Subgroups**																								
Sex																								
Men	59.8	15.2	***	55.8	18.1	***	61.0	19.8	*	63.0	14.2	**	61.7	14.2	***	57.4	18.1	***	59.0	17.9	**	70.2	14.3	***
Women	49.9	13.1		42.5	17.1		48.9	17.9		59.8	11.3		55.2	12.2		49.0	16.2		52.9	15.4		64.4	13.5	
Age																								
< 50 years	55.0	15.2		49.5	18.8		55.3	20.0		60.7	12.5		58.2	14.1		53.4	18.6		56.4	16.7		65.1	14.6	*
≥ 50 years	53.7	14.7		47.3	18.6		53.3	19.4		61.6	12.8		57.9	13.0		52.0	16.8		55.0	16.8		68.3	13.6	
Marital status																								
Single	56.5	14.7	*	51.8	17.5	**	56.0	20.2		62.1	12.1		61.1	13.9	**	55.6	18.3	*	58.3	16.6		70.0	14.8	**
Married	53.7	14.9		47.3	19.0		53.6	19.5		61.0	12.9		57.2	13.2		51.8	17.3		54.9	16.8		66.1	13.9	
Household income																								
< 5 million yen	50.5	14.1		43.4	17.8		49.2	19.1		60.7	13.7		58.3	13.1		52.6	17.4		56.4	16.2		67.1	13.9	
≥ 5 million yen	56.9	14.9		51.6	18.7		57.5	19.4		61.5	11.9		57.8	13.7		52.7	17.7		55.0	17.2		66.8	14.3	
Educational attainment																								
≤ High school	49.1	15.6	*	42.1	19.5		46.4	20.0	**	60.9	14.0		58.5	14.5		51.4	18.3		56.6	17.4		68.7	14.6	
≥ Two years of college	57.1	13.7		51.6	17.5		58.3	18.2		61.4	11.9		57.8	12.9		53.3	17.2		55.0	16.4		65.9	13.8	
Employment status																								
Full-time	57.1	14.8		52.1	18.1		58.2	19.2	**	61.4	12.8		59.2	13.7		54.0	18.0		56.7	17.3		67.8	14.2	
Part-time	45.3	11.3		36.2	15.5		41.4	15.4		60.7	12.5		54.4	11.9		48.2	15.5		52.2	14.5		64.1	13.6	
Workstyle																								
Desk-based	62.3	10.6	***	58.5	12.8	***	64.9	13.6	***	62.7	11.7	*	59.5	14.0		55.2	17.5	*	56.9	17.3		67.5	15.0	
Non-desk-based	41.4	11.2		31.9	14.6		36.9	14.9		58.8	13.8		55.7	12.2		48.5	17.0		53.6	15.7		66.0	12.7	
Body Mass Index																								
< 25	53.0	14.4		46.7	18.2		52.5	19.4		60.9	12.6		57.4	13.1		51.6	16.9		54.8	16.6		66.7	13.8	
≥ 25	60.5	15.7		56.1	19.8		62.4	19.2		63.0	13.1		61.3	14.8		57.9	20.1		59.5	17.4		68.2	15.7	
Smoking status																								
Smokers	55.0	14.7		49.8	18.6		54.7	20.0		61.7	12.9		61.1	14.6		56.4	18.0		57.9	18.9		69.5	15.5	
Non-smokers	54.1	14.9		48.0	18.8		54.0	19.6		61.1	12.7		57.5	13.2		52.0	17.4		55.2	16.4		66.5	13.9	
Alcohol consumption																								
≤ 1–3 times/month	52.5	14.9		45.9	18.9		51.0	19.5		61.7	12.5		58.8	14.1	*	54.4	18.0	***	55.4	17.4		67.4	14.9	
≥ 1 time/week	56.0	14.7		50.7	18.3		57.2	19.4		60.8	12.9		57.3	12.8		50.8	17.0		55.8	16.2		66.5	13.4	
Residential area																								
Matsuyama	50.2	14.5	**	43.2	18.9	**	48.6	18.3	**	60.8	13.4		58.4	13.5		52.5	17.8		56.0	16.9		67.6	14.1	
Koto	58.1	14.3		53.2	17.3		59.4	19.5		61.6	12.0		57.7	13.5		52.8	17.4		55.1	16.7		66.3	14.2	
***Breaks per sedentary hour***																								
**All participants**	9.9	3.2		12.8	6.2		11.0	5.2		8.1	3.1		8.6	3.4		10.4	5.5		9.6	5.1		7.3	3.7	
**Subgroups**																								
Sex																								
Men	8.9	3.3	***	10.5	5.5	***	9.3	4.8	***	8.3	3.7		8.0	3.3	***	9.3	4.8	***	9.0	5.2	*	7.0	3.8	
Women	10.7	2.9		14.6	6.1		12.3	5.1		8.0	2.6		9.1	3.4		11.4	5.8		10.1	5.0		7.5	3.6	
Age																								
< 50 years	10.0	3.4		12.6	6.3		10.9	5.4		8.4	3.2		8.9	3.6		10.9	6.2		9.5	4.7		7.9	3.9	**
≥ 50 years	9.9	3.1		13.0	6.1		11.1	5.0		7.9	3.1		8.4	3.2		10.1	4.8		9.7	5.4		6.8	3.5	
Marital status																								
Single	9.3	3.0	**	11.6	4.9	**	10.7	5.6		7.6	3.0		7.8	3.5	**	9.6	5.7	*	8.8	5.0		6.4	3.2	**
Married	10.1	3.2		13.2	6.4		11.1	5.1		8.3	3.2		8.8	3.3		10.7	5.4		9.9	5.1		7.5	3.8	
Household income																								
< 5 million yen	10.3	2.9		13.8	6.0		11.9	5.2		7.9	3.1		8.5	3.3		10.6	5.9		9.4	4.8		7.0	3.6	
≥ 5 million yen	9.7	3.4		12.2	6.3		10.4	5.1		8.3	3.1		8.7	3.5		10.4	5.2		9.8	5.3		7.5	3.8	
Educational attainment																								
≤ High school	10.5	3.1		14.3	6.8		12.4	5.2		7.9	3.2		8.3	3.7		10.4	5.8		9.2	5.5		6.8	3.6	
≥ Two years of college	9.6	3.2		12.0	5.7		10.3	5.0		8.2	3.1		8.8	3.2		10.5	5.3		9.9	4.9		7.6	3.8	
Employment status																								
Full-time	9.6	3.3		12.0	6.0		10.3	5.0		8.2	3.3		8.4	3.5		10.2	5.6		9.5	5.3		7.2	3.8	
Part-time	11.0	2.6		15.5	5.9		13.2	5.0		7.8	2.6		9.1	3.1		11.2	4.9		10.0	4.4		7.7	3.5	
Workstyle																								
Desk-based	8.8	3.0	***	10.2	4.3	***	9.0	4.1	***	8.1	3.1		8.4	3.6		9.9	5.2		9.5	5.3		7.3	4.0	
Non-desk-based	11.7	2.7		17.0	6.4		14.2	5.1		8.2	3.2		8.9	3.0		11.4	5.8		9.9	4.8		7.3	3.3	
Body Mass Index																								
< 25	10.2	3.1	*	13.2	6.0		11.5	5.1	*	8.2	3.1		8.7	3.3		10.6	5.2		9.8	5.2		7.3	3.5	
≥ 25	8.6	3.2		10.7	6.5		8.9	4.8		7.9	3.4		8.1	3.9		9.9	6.8		8.9	4.9		7.1	4.6	
Smoking status																								
Smokers	10.0	3.3		12.5	6.3		10.5	4.5		9.0	3.7	*	8.2	3.3		9.8	5.5		9.3	5.1		6.9	3.5	
Non-smokers	9.9	3.2		12.9	6.2		11.1	5.3		8.0	3.0		8.7	3.4		10.6	5.5		9.7	5.1		7.4	3.8	
Alcohol consumption																								
≤ 1–3 times/month	10.2	3.0		13.7	6.6		11.7	5.2		7.9	2.8		8.6	3.6		10.3	5.8		9.9	5.5		7.3	3.9	
≥ 1 time/week	9.7	3.3		12.0	5.6		10.4	5.1		8.4	3.4		8.6	3.1		10.6	5.0		9.4	4.8		7.3	3.6	
Residential area																								
Matsuyama	10.8	3.1	***	14.6	6.6	***	12.4	5.1	***	8.3	3.4		8.6	3.4		10.8	5.8		9.6	5.2		7.1	3.5	
Koto	9.1	3.1		11.1	5.1		9.7	4.9		8.0	2.9		8.6	3.4		10.2	5.1		9.6	5.1		7.5	3.9	

LPA, light-intensity physical activity; MVPA, moderate-to-vigorous intensity physical activity; SB, sedentary behavior; SD, standard deviation. Using multiple regression, the differences in the measures of the proportion of sedentary time and number of breaks between subgroups of each sociodemographic variable were examined, adjusting for other sociodemographic variables. *** *p* < 0.001, ** *p* < 0.01, * *p* < 0.05.

## Supplementary Materials

The following are available online at https://www.mdpi.com/1660-4601/17/11/3814/s1, Supplementary Table: Characteristics of objectively measured sedentary behavior and physical activity.
