# Supplementary material for: Accelerometer-Measured Diurnal Patterns of Sedentary Behavior among Japanese Workers: A Descriptive Epidemiological Study
Source: Int J Environ Res Public Health. 2020 May 27;17(11):3814. doi: 10.3390/ijerph17113814 (PMC7312996; doi:10.3390/ijerph17113814)
Supplement: Supplementary file 1 [file ijerph-17-03814-s001.pdf]

Supplementary Table. Characteristics of objectively measured sedentary behavior and physical activity

	Workdays		Non-workdays	
	Mean	SD	Mean	SD
Accelerometer data				
Wear time (minutes)				
Morning	307.5	44.9	261.5	62.9
Afternoon	355.2	15.1	344.8	33.4
Evening	274.3	52.8	274.2	62.8
SB (minutes)				
Morning	146.6	58.6	135.2	50.3
Afternoon	192.5	71.0	191.6	60.7
Evening	168.2	48.3	183.9	58.9
LPA (minutes)				
Morning	129.4	57.2	105.3	49.5
Afternoon	134.9	59.2	128.3	52.3
Evening	88.0	35.6	79.6	39.1
MVPA (minutes)				
Morning	31.5	23.2	21.1	18.9
Afternoon	27.8	22.6	24.9	19.2
Evening	18.1	13.8	10.7	10.2

LPA, light-intensity physical activity; MVPA, moderate-to-vigorous intensity physical activity; SB, sedentary behavior; SD, standard deviation.
